# A Case of Fournier’s Gangrene: A Rare, Lethal Skin Infection

**DOI:** 10.7759/cureus.44383

**Published:** 2023-08-30

**Authors:** Aditi Chokshi, Laura Ziton

**Affiliations:** 1 Dermatology, Nova Southeastern University Dr. Kiran C. Patel College of Osteopathic Medicine (KPCOM), Fort Lauderdale, USA; 2 Family Medicine, Northwest Medical Center, Margate, USA; 3 Family Medicine, Broward Health, Coral Springs, USA; 4 Family Medicine, Dr. Kiran C. Patel College of Osteopathic Medicine (KPCOM), Fort Lauderdale, USA

**Keywords:** dermatology, skin exam, infectious, urology training, fournier gangrene

## Abstract

Fournier’s gangrene (FG) is a rare form of necrotizing fasciitis that is characterized by fascial necrosis of the genitalia or perineum. FG typically results as a complication of genital or anorectal abscess, pressure sore, or surgical site infections. Many patients present with no symptoms, whereas other patients may present with non-specific symptoms such as pain or erythema in the genital or perianal regions. We present a case of FG in a 76-year-old male. Our patient presented initially with only complaints of perianal and groin pain. Upon imaging and skin examination, a diagnosis of Fournier’s gangrene was made. However, due to the late recognition and treatment of FG, the patient developed a sequence of fatal complications that ultimately resulted in his passing. This case demonstrates the importance of a rapid diagnosis of this rare disease to prevent fatal complications. We hope to inform dermatologists, internists, and urologists of the varying presentations of Fournier’s gangrene to allow for prompt initiation of treatment.

## Introduction

Fournier’s gangrene (FG) is a rare form of necrotizing fasciitis characterized by fascial necrosis of the genitalia or perineum with an incidence of only 1.6 cases per 100,000 males [1]. The median age of patients affected by FG is 50.9 years, with the ratio of men to women being 10:1 [2]. Underlying bacteremia initiates the inflammatory mechanism that results in the development of FG. The cytokine cascade activation results in a damaged endothelium, activation of the coagulation cascade, and extravasation of blood. This further leads to swelling of the tissues, leukocyte infiltration, and ultimately, ischemic facial necrosis [2]. FG is a polymicrobial infection caused by both aerobic and anaerobic species with Proteus mirabilis being the most common [3]. These bacterial organisms release collagenases into the tissue, which results in rapid tissue destruction and necrosis, resulting in the infection to disseminate from the genital/perianal region to vital organs and the abdominal wall [4]. Organisms may act directly at the lesion site or disseminate into the circulatory system, resulting in severe toxicity, and organ hypoperfusion, potentially leading to sepsis [5]. FG typically results as a complication of genital or anorectal abscess, pressure sore, or surgical site infections [6]. FG has a variable presentation, with 40% of patients presenting asymptomatically. Early symptoms may include pain or erythema in the genital or perianal regions. Progressive symptoms of crepitus, malodorous discharge, and skin color change occur as the infection infiltrates the deep fascial planes [4].

However, patients with diabetes mellitus, HIV, or morbid obesity have increased susceptibility to the development of severe Fournier’s gangrene [7]. These patients may have an abnormal subacute presentation with generalized symptoms such as fever and fatigue. In these patients, a high index of suspicion is imperative to allow for the early recognition and rapid initiation of treatment. This case highlights the importance of an early dermatologic exam, as FG has many variable presentations with skin changes being the earliest manifestations. Equipping clinicians with knowledge of risk factors, anatomy, and common causes of FG allows for prompt diagnosis given the high mortality rates associated with delayed treatment.

## Case presentation

**Figure 1:**
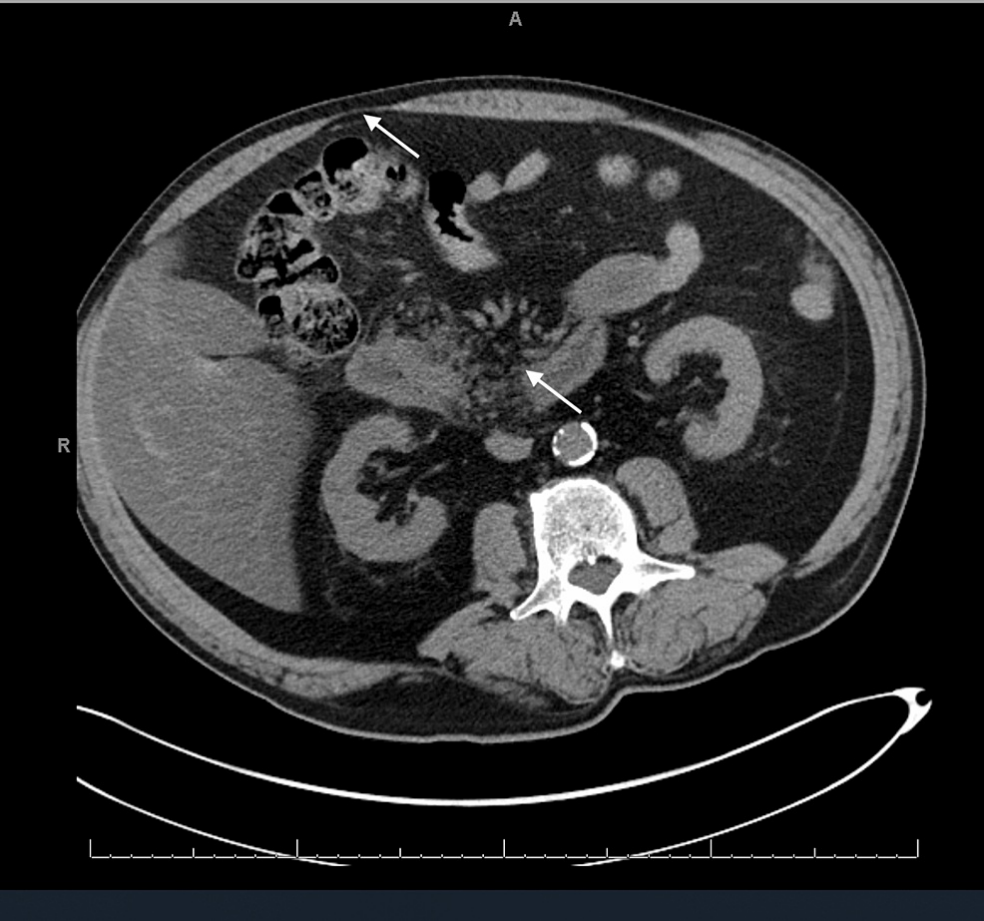
CT scan of pelvis

A 76-year-old presented to the emergency department with complaints of perianal and groin pain. He had a history of nocturia, diabetes mellitus, and heavy smoking history. Additionally, the patient had symptoms of drug overdose, drug abuse, and development of alcohol withdrawal upon admission. Upon admission, dermatological examination revealed foul-smelling black necrotic skin surrounded by patches of erythema and edema. A computed tomography scan showed severe inflammation of the perineum and extensive subcutaneous air on the left side consistent with Fournier’s gangrene (Figure 1).

**Figure 2:**
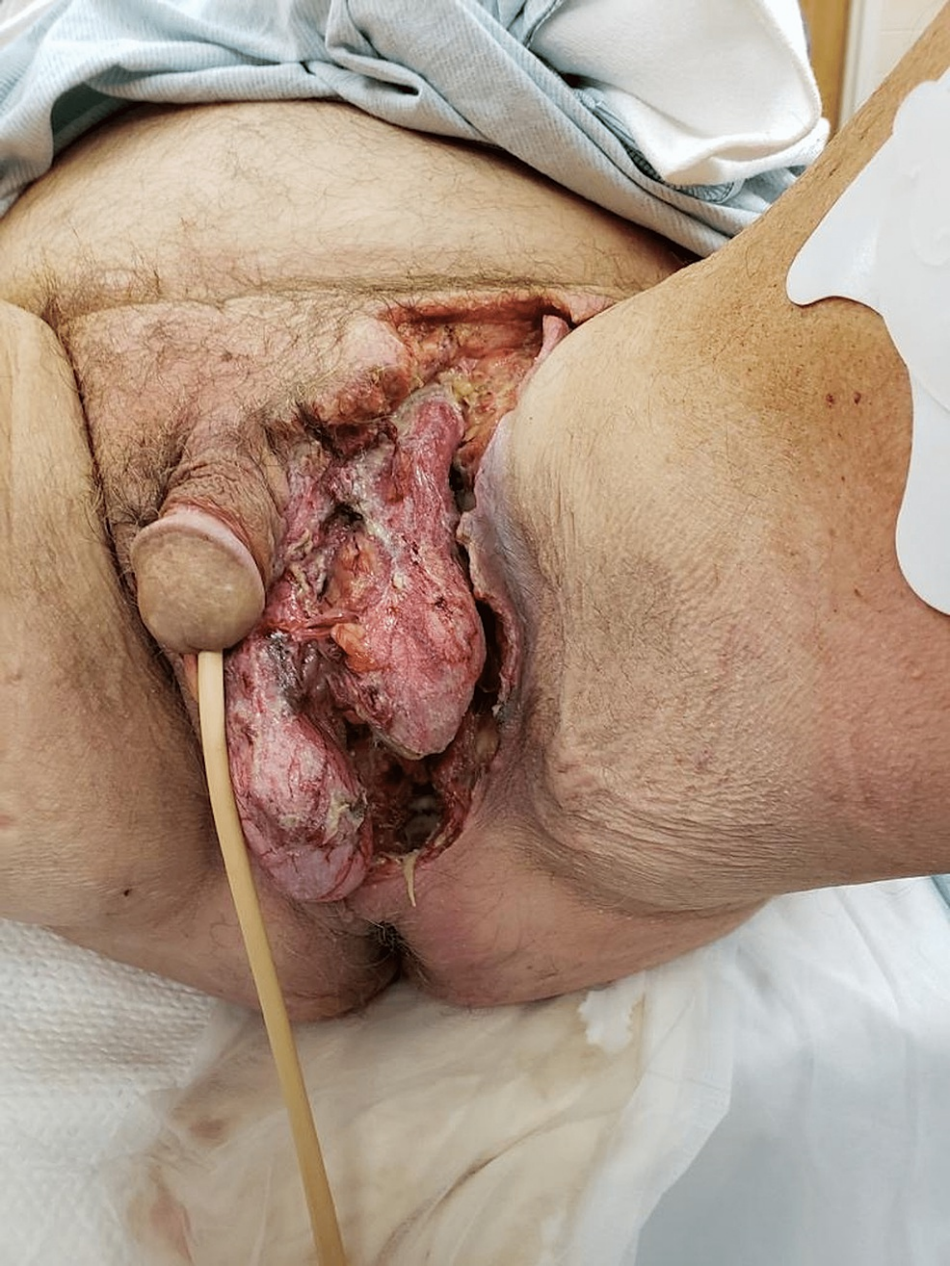
Gangrenous process spread to left groin and perineum

Laboratory evaluation showed the patient to have severe sepsis with leukocytosis of 18.75 *103 and mild lactic acidosis. IV Zosyn and clindamycin were initiated promptly. The patient underwent urgent scrotal exploration and debridement of the gangrenous tissue and the necrotizing fasciitis of the perianal and inguinal areas as seen in Figure 2. 

Surgical pathology confirmed the diagnosis of Fournier’s gangrene showing multiple portions of mottled brown necrotic soft tissue and keratotic skin. Seventy-two hours later, the patient underwent another extensive surgical debridement and placement of a wound vacuum. His cultures grew Morganella and Klebsiella, which resulted in the alteration of antibiotic therapy to meropenem, doxycycline, and micafungin. On postoperative day 7, he was noted to have maroon-colored stools and severe anemia requiring two blood transfusions. Esophagogastroduodenoscopy confirmed the presence of severe gastric/duodenal ulcerations. The patient then further deteriorated with the development of altered mental status, encephalopathy, and a large aspiration episode, resulting in myocardial infarction and respiratory failure. The patient was intubated, and after five days, failed to improve. The family of the patient made the decision to withdraw artificial life support and admit the patient to hospice for palliative care.

## Discussion

Despite advances in surgical techniques and modern medicine, the mortality rate of FG is still high due to delays in diagnosis and treatment. This case illustrates the need for prompt recognition of dermatologic findings, such as erythema, edema, and necrotic tissue, to avoid the development of complications as seen in this case. The severity of FG can be masked since the extent of hypodermic necrosis is greater than can be visualized at a superficial level and progresses rapidly [8]. Observation of erythema that extends superiorly over the inguinal areas should raise suspicion in patients with comorbidities such as diabetes, HIV, or morbid obesity [9]. Our patient had several risk factors, such as diabetes, heavy smoking history, and drug abuse, which should have raised clinical suspicion earlier. Additionally, cultures growing Klebsiella should raise suspicion for STDs such as donovanosis, which may mimic FG. Donovanosis causes granuloma inguinale, which may initially present similarly with foul-smelling, painful, necrotic ulcers [1]. Additionally, the cutaneous signs of FG are also difficult to differentiate from cellulitis erysipelas. Rapid progression of cutaneous lesions, poor therapeutic responses to medications, and systemic signs can help point dermatologists toward the diagnosis of FG [10]. Various imaging modalities may be utilized to diagnose Fournier's gangrene such as X-ray, ultrasound, computed tomography (CT), and magnetic resonance imaging (MRI). If subcutaneous air or gas is discovered on imaging, such as in this patient, this is an indication for immediate urgent surgical exploration. The laboratory risk indicator for necrotizing fasciitis (LRINEC) may also be utilized as an additional diagnostic aid. This score takes parameters such as hemoglobin, white cell count, sodium, creatinine, glucose, and C-reactive protein into account to quantify and stratify the risk of the development of FG [11]. Although radiological studies and laboratory investigations can aid in diagnosis, a thorough skin exam remains the first line that leads to expedited treatment [12]. Treatment with rapid aggressive surgical debridement, early initiation of IV antibiotics, and close monitoring can help reduce morbidity and mortality rates and improve patient outcomes [13]. A 2007 study demonstrated that rapid, aggressive debridement resulted in a significant reduction in mortality, of up to 16% [14]. If surgical debridement is unable to be performed, clinicians may begin treatment with broad-spectrum antibiotics along with plasma exchange as a second-line treatment. This serves to improve organ function and reduce the levels of inflammatory mediators in the bloodstream [5]. Additionally, the use of alternative therapies, such as hyperbaric oxygen and vacuum-assisted closure, remains controversial within the current literature. More research is needed to further evaluate the efficacy of alternative treatment options for patients who are not surgical candidates.

## Conclusions

We report a 76-year-old male who presented with perianal and groin pain. FG is known to have varying subacute presentations. Patients with diabetes mellitus, HIV or morbid obesity have an increased propensity to develop severe FG and fatal complications. In these patients, a high index of suspicion is imperative to allow for the early recognition and rapid initiation of treatment. This case demonstrates the importance of keeping FG on the differential to allow for the early diagnosis and rapid initiation of treatment and debridement.
